# Bronchoalveolar Lavage Fluid Reflects a T_H_1-CD21^low^ B-Cell Interaction in CVID-Related Interstitial Lung Disease

**DOI:** 10.3389/fimmu.2020.616832

**Published:** 2021-02-05

**Authors:** David Friedmann, Susanne Unger, Baerbel Keller, Mirzokhid Rakhmanov, Sigune Goldacker, Gernot Zissel, Björn C. Frye, Jonas C. Schupp, Antje Prasse, Klaus Warnatz

**Affiliations:** ^1^ Divison of Immunodeficiency, Department of Rheumatology and Clinical Immunology, Medical Center—University of Freiburg, Faculty of Medicine, University of Freiburg, Freiburg, Germany; ^2^ Center for Chronic Immunodeficiency (CCI), Medical Center—University of Freiburg, Faculty of Medicine, University of Freiburg, Freiburg, Germany; ^3^ Faculty of Biology, University of Freiburg, Freiburg, Germany; ^4^ Institute of Experimental Immunology, University of Zurich, Zurich, Switzerland; ^5^ Center for Human Genetics and Laboratory Diagnostics (AHC), Martinsried, Germany; ^6^ Department of Pneumology, University Medical Center—University of Freiburg, Faculty of Medicine, University of Freiburg, Freiburg, Germany; ^7^ Pulmonary, Critical Care and Sleep Medicine, Yale University School of Medicine, New Haven, CT, United States; ^8^ Department of Respiratory Medicine, Hannover Medical School and Biomedical Research in End-stage and Obstructive Lung Disease Hannover, German Lung Research Center (DZL), Hannover, Germany; ^9^ Fraunhofer Institute for Toxicology and Experimental Medicine, Hannover, Germany

**Keywords:** common variable immunodeficiency, interstitial lung disease, cytokines, CD21low B cells, T_FH_ and T_PH_ cells

## Abstract

**Background:**

About 20% of patients with common variable immunodeficiency (CVID) suffer from interstitial lung disease (ILD) as part of a systemic immune dysregulation. Current understanding suggests a role of B cells in the pathogenesis based on histology and increased levels of BAFF and IgM associated with active disease corroborated by several reports which demonstrate the successful use of rituximab in CVID-ILD. It is debated whether histological confirmation by biopsy or even video-assisted thoracoscopy is required and currently not investigated whether less invasive methods like a bronchoalveolar lavage (BAL) might provide an informative diagnostic tool.

**Objective:**

To gain insight into potential immune mechanisms underlying granulomatous and lymphocytic interstitial lung disease (GLILD) and to define biomarkers for progressive ILD by characterizing the phenotype of B- and T-cell populations and cytokine profiles in BAL fluid (BALF) of CVID-ILD compared to sarcoidosis patients and healthy donors (HD).

**Methods:**

Sixty-four CVID, six sarcoidosis, and 25 HD BALF samples were analyzed by flow cytometric profiling of B- and T-cells and for cytokines by ELISA and Multiplexing LASER Bead technology.

**Results:**

Both sarcoidosis and CVID-ILD are characterized by a predominantly T-cell mediated lymphocytosis in the BALF. There is an increase in T follicular helper (T_FH_)-like memory and decrease of regulatory T cells in CVID-ILD BALF. This T_FH_-like cell subset is clearly skewed toward T_H_1 cells in CVID-ILD. In contrast to sarcoidosis, CVID-ILD BALF contains a higher percentage of B cells comprising mostly CD21^low^ B cells, but less class-switched memory B cells. BALF analysis showed increased levels of APRIL, CXCL10, and IL-17.

**Conclusion:**

Unlike in sarcoidosis, B cells are expanded in BALF of CVID-ILD patients. This is associated with an expansion of T_FH_- and T_PH_-like cells and an increase in APRIL potentially supporting B-cell survival and differentiation and proinflammatory cytokines reflecting not only the previously described T_H_1 profile seen in CVID patients with secondary immune dysregulation. Thus, the analysis of BALF might be of diagnostic value not only in the diagnosis of CVID-ILD, but also in the evaluation of the activity of the disease and in determining potential treatment targets confirming the prominent role of B-cell targeted strategies.

## Introduction

Common variable immunodeficiency (CVID) is an antibody deficiency syndrome (www.esid.org) with a heterogeneous, mostly unknown pathogenesis. This most common primary immunodeficiency is defined by reduction of serum IgG, IgA, and/or IgM and impaired antibody responses together with disturbed memory B cell and plasma cell development (1, 2). Mutations in several genes have been associated with the clinical presentation of CVID, currently explaining only less than 20% of CVID cases (3, 4). Clinically, most CVID patients suffer from recurrent bacterial infectious diseases, particularly of the respiratory tract. This is frequently associated with the development of bronchiectasis over time (5). Additionally, around 50% of CVID patients have secondary noninfectious lymphoproliferative, autoimmune and inflammatory complications like autoimmune cytopenias, granulomatous disease, splenomegaly and lymphadenopathy, interstitial lung disease, enteropathy and hepatopathy (6) often contributing to a significantly reduced quality of life and increased morbidity and mortality (7–10).

Interstitial lung disease (CVID-ILD) is one of the main complications in CVID. It manifests in about 20% of CVID patients and may be present already at the initial diagnosis in a relevant subgroup of patients frequently leading to the misdiagnosis of sarcoidosis (11, 12). No infectious agent has been reliably identified as a trigger of the disease and CVID-ILD is felt to be part of the systemic lymphoproliferative immune dysregulation. It manifests variably with follicular bronchiolitis, lymphocytic interstitial pneumonia and nodular mostly granulomatous lung disease (13–15). Maglione et al. described B cell containing tertiary lymphoid germinal center (GC)-like structures within the affected lung tissue (16). Recently, they suggested that active CVID-ILD is driven by pulmonary B cell hyperplasia which is reflected by elevated BAFF-mediated apoptosis resistance and an increase in serum IgM (17). The pivotal role of B cells in the lung pathology is underpinned by the positive effect of B-cell depleting therapies on CVID-ILD (18).

The optimal form of treatment has however not yet been defined. IgG replacement therapy alone rarely prevents or improves CVID-ILD (15, 19, 20), thus immunosuppressive therapy is frequently used to control the pulmonary manifestations of the immune dysregulation (21).

Diagnosis is currently often based on CT morphology and pulmonary function tests (22, 23) with no additional histological or other confirmation. The need for confirmation by video-assisted thoracoscopic surgery (VATS) assisted lung biopsies is postulated by some (13), but not endorsed by others due to the invasive character of the procedure and the lack of significant impact on diagnosis in the majority of cases (24).

Therefore, we set out to retrospectively analyze the data of bronchoalveolar lavage (BAL) in patients with CVID as a less invasive procedure. The patients were seen at the Center for Chronic Immunodeficiency (CCI) in the years between 2004 and 2020.

## Methods

### Patients and BAL Samples Processing

All patients fulfilled the criteria for CVID according to the European Society for Immunodeficiencies (ESID) (www.esid.org) and suffered from interstitial lung disease as determined by radiological and/or lung function abnormalities. The following clinical data was recorded (**Supplementary Table 1**): splenomegaly (defined as a diameter of greater than 11x4.7 cm proven by ultrasound or computer tomography (CT scan); generalized lymphadenopathy (LNs >1 cm in diameter in at least two different anatomical sites detected by clinical examination, ultrasound, or CT); autoimmune cytopenias (autoimmune hemolytic anemia or immune thrombocytopenia); enteropathy (based on clinical presentation, endoscopic analysis and histology when available), liver disease (based on clinical parameters, ultrasound, serum parameters and histology when available). In addition, all patients were classified according to EUROclass classification (25), considering the reduction of switched memory B cells (smB) and the expansion of CD21^low^ B cells.

All procedures performed in this study were in accordance with the ethical standards of the institutional (FR 189/12_120543) research committee and with the 1964 Helsinki declaration and its later amendments. Informed consent was obtained from all individual participants before inclusion into the study. Patients underwent bronchoscopy as part of clinical work-up, i.e. differential diagnosis of respiratory complaints and/or radiological abnormalities. BAL samples were obtained from 64 CVID patients (33 female and 31 male patients, age 17–73 years), 6 sarcoidosis patients (one female and five male patients, age 29 to 76 years and 25 healthy adult volunteers (12 female and 13 male, age 19–67 years). Five former smokers and three smokers could be identified (see **Supplementary Table 1**). BAL samples of diagnostic bronchoscopy were analyzed by the routine laboratory for overall cell counts, vitality, lymphocytes, T cells (including CD4 and CD8 T cell subsets), macrophages, neutrophils, eosinophils and basophils/mast cells as described by Frye et al (26). and the guidelines of the European Respiratory Society (27). Additional phenotyping of T and B cell subsets and cytokine production was performed *via* our research laboratory. Due to the retrospective character, not all investigations were performed from the same samples.

### Immunophenotyping by Using Flow Cytometry

Cells from bronchoalveolar lavage were washed in Iscove’s Modified Dulbecco’s Medium (IMDM) or Roswell Park Memorial Institute (RPMI) media with 10% FCS and further processed for flow cytometry.

B-cell populations were characterized by staining for IgD, IgA, IgM, IgG, CD19, CD21, CD27 and CD38 expression and T cell subsets by their expression of CD3, CD4, CD8a, CD25, CD27, CD28, CD45, CD45RA, CCR6, CXCR3, CXCR5, PD-1, FoxP3, CTLA-4.

All applied antibodies and their vendors are listed in **Supplementary Table 2** in the Online Repository.

Data acquisition was performed on a Gallios flow-cytometer (Beckman Coulter, Miami, FL) or LSR Fortessa (BD Biosciences, Franklin Lakes, NJ). Data were analyzed using FlowJo software (Treestar, Ashland, OR).

### Cytokine Levels in BALF

IL-4, IL-10, IL-12, IL-17, and CXCL10 (IP10) in BALF were analyzed by multiplex bead technology assays using the Luminex^®^ xMAP^®^ platform performed by Eve Technologies Corporation, Calgary, Alberta, Canada.

APRIL, BAFF, CXCL9, CXCL13, CXCL14, and CXCL10 in cell-free BALF were quantified using DuoSet ELISA Kits (R&D Systems) according to the manufacturer’s protocol. All samples were measured in duplicates.

### Statistical Analysis

Values were expressed as means ± SDs. Statistical significance was assessed by the unpaired T test for datasets with Gaussian distribution, or by the Mann-Whitney test for datasets without Gaussian distribution. The Kruskal-Wallis test or ordinary one-way ANOVA were used for multiple comparisons. Correlation data was assessed by simple correlation test.

Results were analyzed with the help of GraphPad Prism software (version 8.4.2; GraphPad Software, La Jolla, Calif), and p values of less than 0.05 were considered significant.

## Results

### Lymphocytic Bronchoalveolar Lavage Fluid in the Majority of CVID-ILD

**Figure 1:**
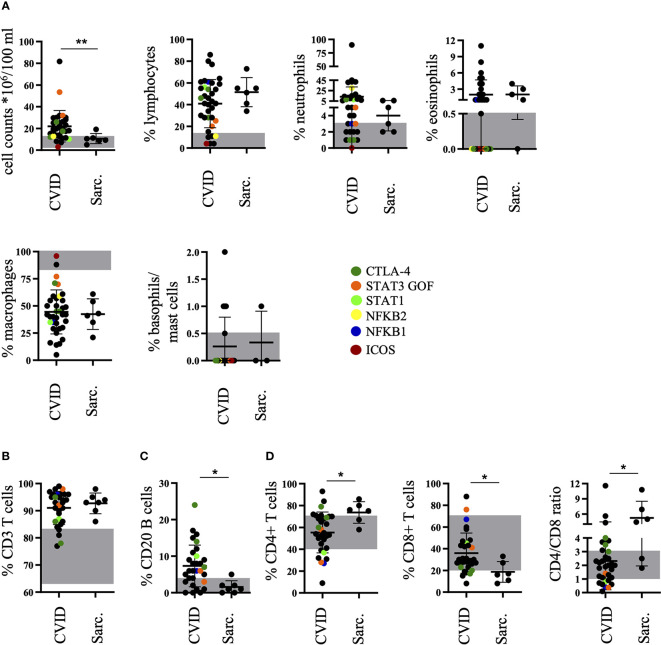
Increased percentage of B cells in bronchoalveolar lavage fluid (BALF) of common variable immunodeficiency (CVID)-interstitial lung disease (ILD) compared to sarcoidosis. The diagnostic workup of the BALF of patients with CVID or sarcoidosis for cell counts, percentages of lymphocytes, neutrophils, eosinophils, macrophages, and basophils/mast cells **(A)**, CD3+ T cells **(B)**, CD20+ B cells **(C)**, as well as CD4+ and CD8+ T cells including CD4/CD8 ratio **(D)**. The normal range is marked in grey for each population and defined genetic defects are marked by color coding. Sarc., sarcoidosis. *P <.05, **P <.01.

The routine diagnostic workup of the BAL samples revealed an increased total cell count. Absolute leukocyte counts were increased in 79% of CVID patients above normal range. These were significantly higher (22.0 × 10^6^/100 ml +/− 14.5 × 10^6^/100 ml) than in the control group with sarcoidosis (10.6 × 10^6^/100 ml +/− 4.7 × 10^6^/100 ml) (**Figure 1A**). In 83% of the CVID patients the analysis revealed an expansion of lymphocytes, 65% of the BALF were characterized by a relative increase in neutrophils and 37% of eosinophils (**Figure 1A**). In 59% of CVID patients, increased neutrophils were associated with the detection of concurrent bacterial or fungal infection. The slight increase in eosinophils could not be attributed to a specific cause and was similarly seen in sarcoidosis. Interestingly, nearly all of the genetically defined immunodeficiencies had no detectable eosinophils. Overall, the cellular composition of the main leukocyte cell differentiation lineages in BALF of CVID-ILD was not significantly different to sarcoidosis.

Also, similar to sarcoidosis, CD3^+^ T cells were increased compared to the normal range in over 90% of CVID patients (**Figure 1B**), but in 67% of CVID patients there was an additional increase of B cells not seen in sarcoidosis (**Figure 1C**). The typically increased CD4/CD8 ratio in sarcoidosis was less frequently seen in CVID patients (**Figure 1D**).

### Expansion of T_FH_ and T_PH_ Cells in BALF of CVID-ILD

**Figure 2:**
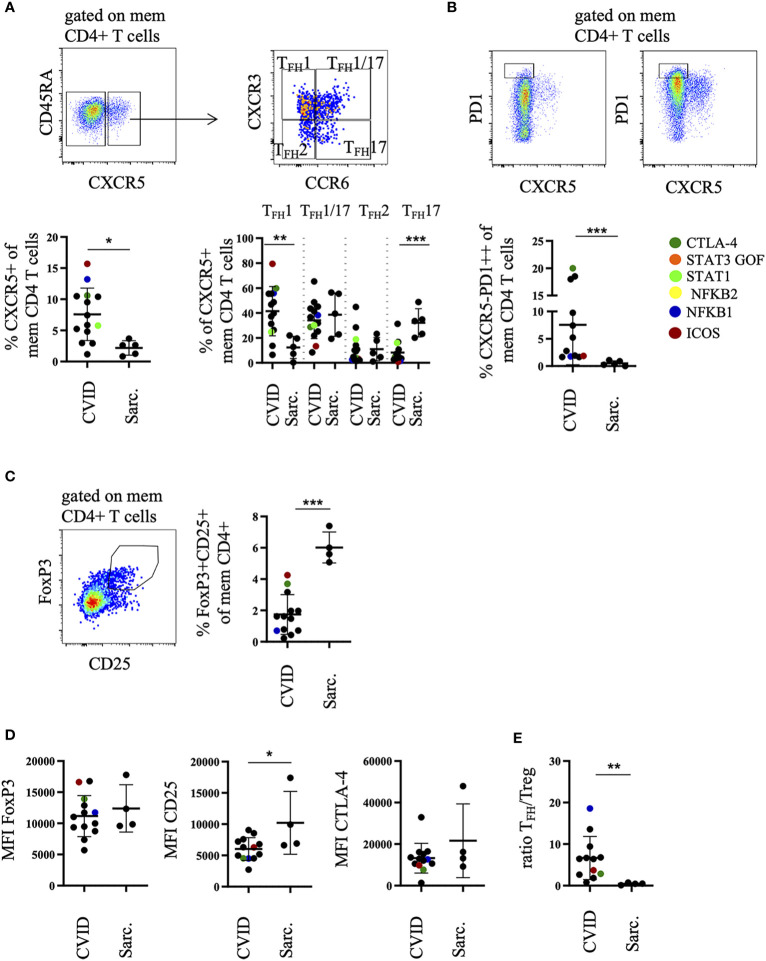
Increased percentage of T_FH_1-like and T_PH_ cells in bronchoalveolar lavage fluid (BALF) of common variable immunodeficiency (CVID)-interstitial lung disease (ILD) compared to sarcoidosis. Memory CD4 T cells were differentiated into CXCR5^pos^ T_FH_1-, T_FH_1/17-, T_FH_17-, T_FH_2-like cell subsets according to their CXCR3 and CCR6 expression **(A)** and total memory CD4 T cells into CXCR5^neg^PD1^high^ T_PH_ cells. Shown are two examples with high and low amounts of T_PH_ cells **(B)**. Corresponding statistics are shown below. Memory CD4 T cells were further differentiated into FoxP3^+^CD25^+^ Tregs, statistics are shown on the right **(C)**. The mean fluorescence intensity (MFI) of FoxP3, CD25, CTLA-4 in Tregs is shown in **(D)** and the ratio of CXCR5^pos^ memory CD4 T_FH_-like cells to Tregs in **(E)**. Defined genetic defects are marked by color coding. *P <.05, **P <.01 ***P <.001, Sarc., sarcoidosis.

Further CD8 T cell phenotyping revealed a similar distribution of effector memory subsets according to their CD27 and CD28 expression compared to patients with sarcoidosis (data not shown). In contrast, additional phenotyping of CD4^+^CD45RA^-^ memory T cells demonstrated an expansion of CXCR5-expressing T follicular helper (T_FH_)-like cells (**Figure 2A**) with a significant increase of CXCR3-expressing T_FH_1-like cells and a decrease of CCR6-expressing T_FH_17-like cells when compared to sarcoidosis (**Figure 2A**). Moreover, there was a significant increase of the recently described CXCR5^neg^PD1^high^ T peripheral helper (T_PH_)-like cell population (28) in BAL samples of CVID patients compared to patients with sarcoidosis (**Figure 2B**).

These changes were associated with a significant decrease of FoxP3^+^CD25^+^ T regulatory cells (Treg) among memory CD4 T cells (**Figure 2C**), expressing lower amounts of CD25 on their surface compared to sarcoidosis patients (**Figure 2D**). As a consequence, the ratio of CXCR5^+^ T_FH_-like cells to Tregs was significantly increased in CVID patients (**Figure 2E**).

We did not detect significant differences in regard to other T-cell populations (data not shown).

### The Expanded B-cell Population Consists Mainly of CD21^low^ B Cells in BALF of CVID-ILD

**Figure 3:**
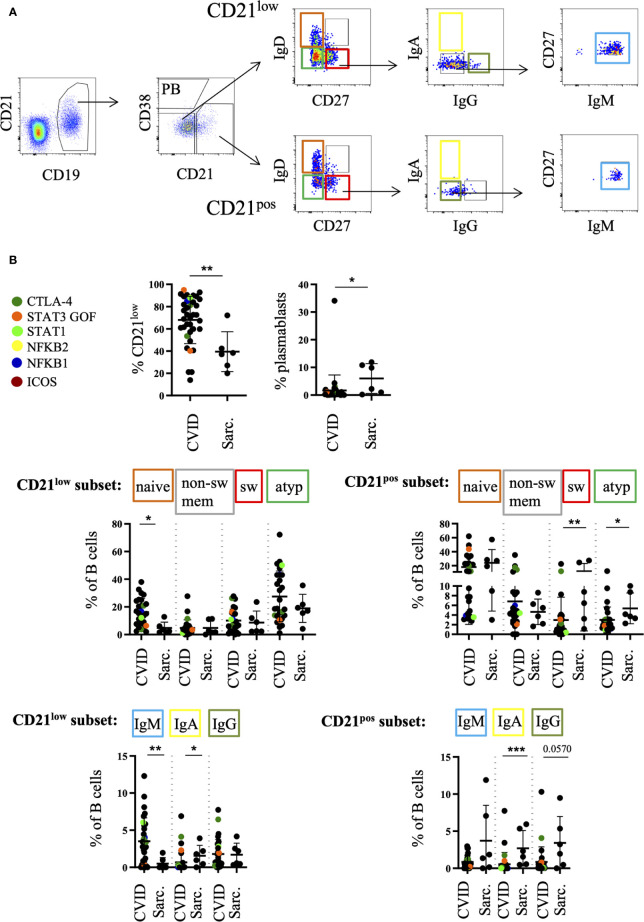
Increased percentage of CD21^low^ cells in bronchoalveolar lavage fluid (BALF) of common variable immunodeficiency (CVID)-interstitial lung disease (ILD) compared to sarcoidosis. B cells were further divided into CD21^low^ B cells, plasmablasts (PB) and CD21^pos^ B cells. An exemplary FACS plot is shown in **(A)**. Naive (IgD^+^CD27^-^), switched memory B cells (IgD^-^CD27^+^), atypical (IgD^-^CD27^-^) and non-switched memory B cells (IgD^+^CD27^+^) were gated from the CD21^low^ B-cell compartment as well as from the CD21+ nonPB subset. IgA, IgG, IgM-only cells were gated out of the switched memory B cell gate (IgD^-^CD27^+^). Corresponding statistical analysis is shown in **(B)**. Defined genetic defects are marked by color coding. *P <.05, **P <.01 ***P <.001, Sarc, sarcoidosis.

Since B cells are expanded in BALF of the majority of CVID patients we investigated their phenotype more closely (**Figure 3A**). As previously reported by our group (29) the main B cell population in the BALF of CVID patients with ILD were CD21^low^ B cells representing T-bet^hi^ B cells (30, 31) (**Figure 3B**). This population was significantly expanded compared to sarcoidosis, while plasmablasts were reduced in the CVID cohort (**Figure 3B**).

The majority of CD21^low^ B cells represented phenotypically as naïve-like CD27^neg^IgD^pos^IgM^pos^ and atypical CD27^neg^IgD^neg^IgM^pos^ B cells (**Figure 3B**). CVID patients differed significantly from sarcoidosis patients in regard to the expansion of their naïve-like B cells within the CD21^low^ compartment as well as the reduction of atypical and switched memory B cells within the CD21^pos^ compartment (**Figure 3B**).

As expected from blood data within the CD27^pos^ memory compartment, CVID patients showed a relative reduction of IgA^pos^ switched memory B cells and increase of IgM-only cells both among CD21^low^ and CD21^pos^ B cells compared to sarcoidosis (**Figure 3B**). Interestingly, especially CD27^pos^ CD21^low^ B cells comprise a comparable amount of IgG^pos^ B cells in the BALF compared to sarcoidosis patients while these cells are usually reduced in peripheral blood of CVID patients (25).

### Increased APRIL, IP10, and IL-17 Concentrations in BALF of CVID-ILD

**Figure 4:**
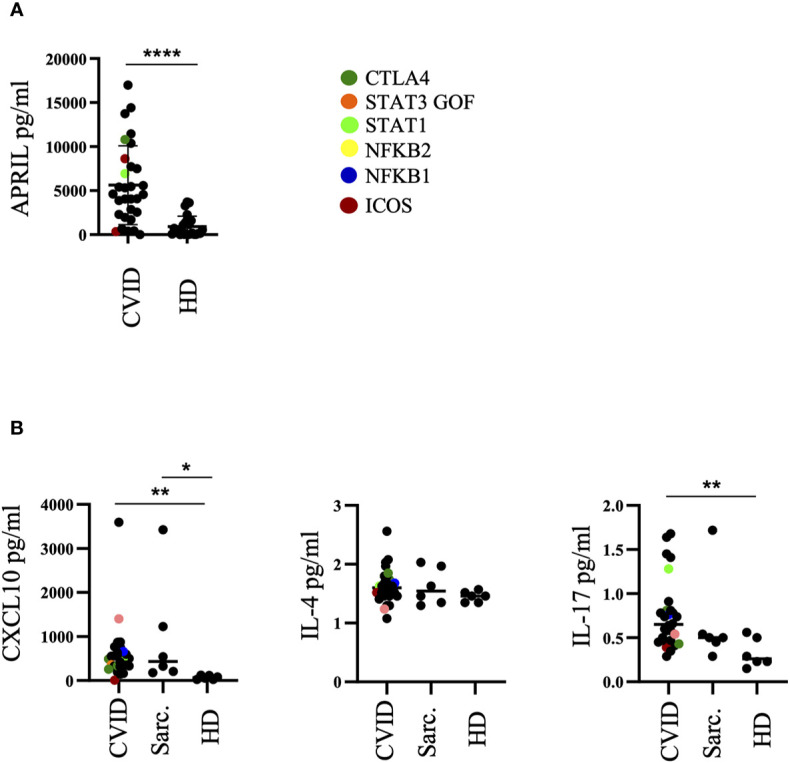
Altered cytokine milieu in bronchoalveolar lavage fluid (BALF) of common variable immunodeficiency (CVID)-interstitial lung disease (ILD). **(A)** ELISA of BALF supernatants for APRIL production. **(B)** Multiplex Bead Array of BALF supernatants for CXCL10, IL-4 and IL-17. Defined genetic defects are marked by color coding. *P <.05, **P <.01 ****P <.0001 HD, healthy control; Sarc, sarcoidosis.

ELISAs of BAL fluids of 30 CVID patients and 25 healthy donors revealed an increased concentration of APRIL in BALF of CVID patients when compared to healthy donors (**Figure 4A**) while BAFF, CXCL9, CXCL13, CXCL14, and CXCL10 (IP10) of the same samples were below the detection limit (data not shown).

In an independent subgroup of CVID patients, sarcoidosis patients as well as healthy donors we performed an analysis by MultiPlex Bead Arrays of BAL fluids for CXCL10, IL-4, IL-10, IL-12, and IL-17. IL-10 and IL-12 of most of the samples were below the detection limit and therefore not shown. CXCL10 and IL-17 concentrations were significantly increased in the BALF of CVID patients compared to healthy donors (**Figure 4B**). CXCL10 was also increased in most of the sarcoidosis patients. No differences were observed for IL-4.

### Correlations Between Cell Subsets and Cytokines in BALF and Peripheral Blood of CVID-ILD

**Figure 5:**
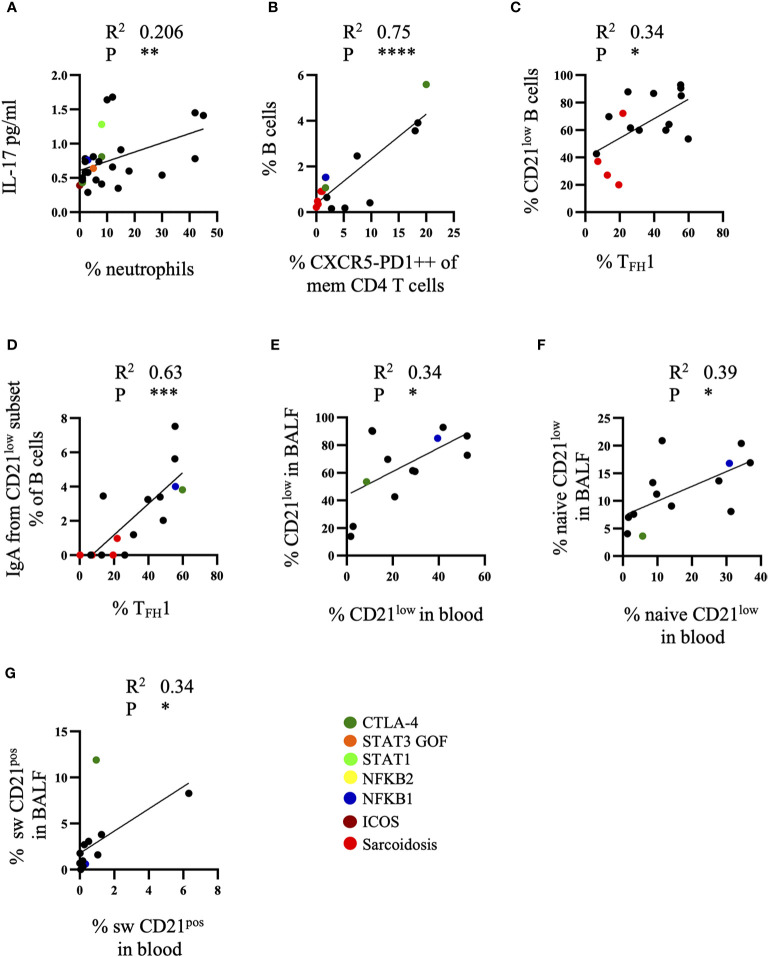
Correlations between cell subsets and cytokines of bronchoalveolar lavage fluid (BALF). **(A)** Correlation of IL-17 in BALF of common variable immunodeficiency (CVID) patients with neutrophil numbers (n = 28), **(B)** of B cells and T_PH_ cells (n = 16), **(C)** of CD21^low^ B cells and T_FH_1 cell subset (n = 16) and **(D)** of IgA^pos^ CD21^low^ B cells and T_FH_1 cells (n = 16). Correlation of total CD21^low^ B cells (n = 13) **(E)**, naïve CD21^low^ B cells (n = 13) **(F)** and switched CD21^pos^ B cells (n = 13) **(G)** in BALF and peripheral blood. Defined genetic defects are marked by color coding. *P <.05, **P <.01 ***P <.001, ****P <.0001.

In order to integrate the different findings we analyzed the association of the accumulation of different cell types and the concentration of the different cytokines and chemokines in BALF. Increased neutrophil counts in BALF of CVID patients positively correlated with elevated levels of IL-17 (**Figure 5A**). We could neither detect a correlation between CXCL10 and the expansion of T_FH_1, T_PH_ cells or CD21^low^ B cells nor of APRIL with total B cells, switched memory B cells or CD21^low^ B cells (data not shown). There was, however, a strong positive correlation of the percentage of B cells and T_PH_ cells in the BALF (**Figure 5B**), and to a lesser degree between the percentage of CD21^low^ B cells and T_FH_1 cells (**Figure 5C**) which originated from a correlation of IgA^pos^ CD21^low^ B cells and T_FH_1 cells (**Figure 5D**). Interestingly, this was not seen for IgG memory B cells.

When comparing the different T and B cell subsets in peripheral blood and BALF of CVID-ILD patients there were not sufficient data of the extended T cell phenotyping for T_FH_ and T_PH_ in peripheral blood performed at the same time in order to draw firm conclusions. When comparing the B-cell subpopulations however there was a significant correlation of the percentage of total (**Figure 5E**) and naïve CD21^low^ B cells (**Figure 5F**) and of switched memory CD21^pos^ B cells (**Figure 5G**) between both compartments.

## Discussion

Interstitial lung disease in patients with CVID is usually characterized by a mixed T- and B-cell infiltrate of the interstitial space (13, 14, 16, 17). Here we could show that this previously reported lymphocytic infiltrate is reflected by the expansion of lymphocytes in the bronchoalveolar space detected in over 80% of the patients. Similar to the histological findings, the majority of the lymphocytes consist of T cells but there is an additional significant expansion of B cells compared to healthy controls and patients with sarcoidosis. Like in peripheral blood, switched memory and especially IgA^pos^ B cells were reduced in BALF of CVID patients compared to sarcoidosis. However, a substantial amount of CVID patients accumulated IgG^pos^ B cells in the BALF despite a profound reduction of IgG^pos^ B cells in blood. As we had previously reported the majority of B cells in the BALF belong to the CD21^low^T-bet^hi^ population (29). Also most of the CD21^low^ B cells which can present as naïve, non-switched and switched classical and atypical memory B cells (31), in the BALF of CVID-ILD had a naïve or non-class switched atypical memory phenotype. This population is linked to a T_H_1 driven inflammatory environment (30) where other costimulatory factors like IL-21 may contribute to their differentiation (32). Compatible with this hypothesis we found an expansion of T_FH_1 cells within the BALF compared to sarcoidosis significantly correlating with the expansion of CD21^low^ B cells, prone to provide both IFN*γ* and IL-21 co-stimulation. Compatible with the role of T_FH_ cells in memory formation, the percentage of T_FH_1 cells demonstrated a highly significant correlation with the percentage of IgA^pos^ memory B cells among the CD21^low^ B-cell population. T_FH_ cells have not been investigated in the bronchoalveolar space before yet our findings support the presence of tertiary GC in the lung tissue of CVID patients with ILD as reported by Maglione et al. (16, 17) and represent a fundamental difference between sarcoidosis- and CVID-associated ILD given that the BALF of the latter not only contain more B cells but also a higher percentage of T_FH_ cells. Corresponding to the low T_FH_ cell proportion in the BALF in sarcoidosis, to our knowledge no tertiary GC formation in the lung has been described in this disease condition. Interestingly, unlike CVID-ILD B cell infiltrates of the inter-granulomatous lung tissue are not reflected in the BALF of sarcoidosis patients (33).

In addition to the relative expansion of T_FH_1 cells reflecting GC activity, there was a significant expansion of the recently discovered T_PH_ cells in BALF of CVID patients with ILD. These cells have a similar capacity like T_FH_ cells in co-stimulation of B cells but are usually found in peripheral tissues without bona fide GC activity. They have been described in the synovium of patients with active rheumatoid arthritis (28), in inflamed intestinal tissues in Crohn’s disease (34), IgG4-related diseases (35, 36), systemic sclerosis (37), IgA nephropathy (38), type I diabetes (39) and most likely within loose lymphocytic aggregates of murine airway inflammation models (40) but also expanded in peripheral blood of rheumatoid arthritis, systemic lupus erythematosus (SLE) and in Sjögren’s syndrome (41–45). Potentially, T_PH_ cells may drive the differentiation of B cells in the less organized inflammatory tissue structures of the lymphocytic infiltrates of the lung (46). Given their capacity for IL-21 and IFN*γ* production T_PH_ cells are good candidates inducing the differentiation of CD21^low^T-bet^hi^ B cells in peripheral tissues. Especially the “atypical memory” CD21^low^ B-cell population as the largest population in BALF of many CVID-ILD patients might be the main target B-cell population of T_PH_ cell interaction in the lung as had been previously suggested in lupus (47–49). We found a highly significant correlation of T_PH_ cells and B cells in the BALF of patients. Similarly, both CD11c^+^CD21^-^CXCR5^-^ B cells and T_PH_ cells were found increased in lupus nephritis tissues (50, 51). Furthermore the frequency of both cell subsets is highly associated in blood of SLE patients (43, 50).

The analysis of cytokines confirmed an environment supporting T_H_1-driven inflammation and B cell survival and expansion. While we could detect only very low levels of BAFF which had previously been described as an important cytokine in the BALF of CVID-ILD patients (17) we detected high levels of APRIL. This factor may not only allow for local B-cell survival but may actually contribute to the differentiation of the detectable class switched memory B cells as it has the capacity to support class switch in mucosal tissues (52). It is tempting to speculate whether relevant ILD is less common in TACI deficient patients (53) despite the presence of lymphoproliferation and autoimmunity, two manifestations predisposing for ILD in CVID. The increased levels of IL-12 in some patients demonstrate a potential bias of non-lymphocytic cells like local macrophages endorsing the T_H_1 environment. Similar to sarcoidosis CXCL10 is significantly elevated in CVID-ILD derived BALF being one of the main chemokines attracting not only CXCR3 positive T_H_1 cells but also CD21^low^T-bet^hi^ B cells which likewise express high levels of this chemokine receptor (30). Interestingly, unlike the gastrointestinal tissue (54) we could also detect elevated IL-17 concentration in some of the CVID-ILD BALF. Given the reduction of T_H_17 cells in the BALF of CVID patients IL-17 must be mainly produced by T_H_1/17 cells. Increased IL-17 concentrations were associated with an increased proportion of neutrophils in the BALF as IL-17 supports their recruitment. This seems to be frequently driven by additional bacterial airway infection.

When comparing the lymphocyte subsets circulating in peripheral blood with the subsets in BALF, we did not have sufficient data on T cell populations in order to draw definite conclusions, but among B cells there was a significant correlation between the percentage of total and naïve CD21^low^ B cells and switched memory CD21^pos^ B cells in both compartments. While the first most likely reflects a direct communication between both pools, we assume that the correlation of the percentage of switched memory B cells rather reflects the general capacity of the patient to class switch. In order to confirm these assumptions, BCR sequencing of both compartments is required in order to determine clonal relationship.

Future studies will also need to perform direct comparison of BALF and histology of lung tissue in order to determine how much the changes we could demonstrate in BALF in this study truly reflect the pathology in the tissue. Such studies will require in depth phenotyping of T and B cells, including TCR and BCR sequencing to demonstrate clonal relationship between the lymphocyte populations, a careful evaluation of the cytokine milieu and foremost the sensitivity of BALF analysis for lymphoma as a differential diagnosis in ILD of CVID.

In summary, BALF of CVID patients with ILD is mainly characterized by an expansion of lymphocytes. Unlike in sarcoidosis these consist of a mixed T- and B-cell expansion reflecting the mixed infiltrates in lung tissue of CVID-ILD patients. The simultaneous expansion of CD21^low^T-bet^hi^ B cells, T_FH_1 and T_PH_ cells in the BALF of CVID-ILD strongly points toward cognate interactions of these populations potentially in tertiary GCs driving the lymphocytic interstitial pneumonitis often seen in these patients. This hypothesis is supported by the cytokine milieu identified in the BALF. Based on these findings it will be of high interest to test whether detailed analysis of BALF sufficiently reflects the pathology of the lung tissue in order to potentially render BALF analysis a valuable tool in diagnosing the presence and activity of ILD in CVID and guide treatment decisions.

## Data Availability Statement

The original contributions presented in the study are included in the article/**Supplementary Material**. Further inquiries can be directed to the corresponding author.

## Ethics Statement

The studies involving human participants were reviewed and approved by Ethics Committee of the University Medical Center Freiburg, Freiburg, Germany. The patients/participants provided their written informed consent to participate in this study.

## Author Contributions

SU, MR, BK, and DF performed experiments and analyzed the data. DF wrote the first draft of the manuscript. SG provided clinical data. JS and AP supervised ELISAs of BALF. GZ, BF, and AP provided BAL samples. KW devised and supervised the study, designed the research, and edited the manuscript. All authors corrected the manuscript. All authors contributed to the article and approved the submitted version.

## Funding

This work was supported by the German Federal Ministry of Education and Research (Grant BMBF 01E01303) and the Deutsche Forschungsgemeinschaft (grant TRR130 P07) to KW. The article processing charge was funded by the Baden-Wuerttemberg Ministry of Science, Research and Art and the University of Freiburg in the funding programme Open Access Publishing.

## Conflict of Interest

The authors declare that the research was conducted in the absence of any commercial or financial relationships that could be construed as a potential conflict of interest.
